# Long noncoding RNA POU6F2-AS1 regulates lung cancer aggressiveness through sponging miR-34c-5p to modulate KCNJ4 expression

**DOI:** 10.1590/1678-4685-GMB-2020-0050

**Published:** 2021-05-14

**Authors:** Xiao-Yan Wu, Yi Xie, Li-Yun Zhou, Yuan-Yuan Zhao, Jing Zhang, Xiu-Feng Zhang, Shuai Guo, Xue-Yan Yu

**Affiliations:** 1 Shandong Chest Hospital, Department of Respiratory Medicine, Jinan, Shandong, China.

**Keywords:** POU6F2-AS1, lung adenocarcinoma, miR-34c-5p, KCNJ4, cell aggressiveness

## Abstract

It has been extensively reported that long noncoding RNAs (lncRNAs) were closely associated with multiple malignancies. The aim of our study was to investigate the effects and mechanism of lncRNA POU6F2-AS1 in lung adenocarcinoma (LADC).The Cancer Genome Atlas (TCGA) and Gene Expression Omnibus (GEO) datasets provided us the information of LADC clinical samples. High-regulation of POU6F2-AS1 was presented in LADC tissues compared with adjacent normal tissues, which was correlated with poor outcome of LADC patients. Functional experiments in Calu-3 and NCI-H460 cells showed that POU6F2-AS1 significantly promoted LADC cell proliferation, colony formation, invasion and migration. Moreover, through online prediction, luciferase reporter assay and Pearson’s correlation analysis, we found that POU6F2-AS1 may act as a competing endogenous RNA (ceRNA) of miR-34c-5p and facilitated the expression of potassium voltage-gated channel subfamily J member 4 (KCNJ4). The promoting effect of cell aggressiveness induced by POU6F2-AS1 was enhanced by KCNJ4, whilst was abrogated due to the overexpression of miR-34c-5p. Collectively, POU6F2-AS1 might function as a ceRNA through sponging miR-34c-5p to high-regulate KCNJ4 in LADC, which indicates that POU6F2-AS1 might be a promising therapeutic target with significant prognostic value for LADC treatment.

## Introduction

Lung cancer is the most frequent diagnosed malignancy (11.6% of the total cases) and the principal cause of cancer death (18.4% of the total cancer deaths) throughout the world (Bray et al., 2018). Non-small-cell lung cancer (NSCLC) is the main type of lung cancer accounting for 85% of lung cancer, which includes lung adenocarcinoma (LADC) and lung squamous cell carcinoma (LSCC) (Yao et al., 2019). LADC is considered as the most common histological subtype of NSCLC (Miyahara et al., 2019). With the increasing prevalence of LADC, it gradually becomes a health challenge for human. In spite of great improvement in the therapeutic therapies has been made, the prognosis of LADC patients remains disappointing with less than 20% survival rates (Kumar Shah et al., 2017; Saad et al., 2017; Drake et al., 2018). Thereby, extensive explorations are urgently needed to unearth the novel effective targets for the treatment of LADC.

Potassium voltage-gated channel subfamily J member 4 (KCNJ4) is classified as a member of the inward rectifier potassium channel family, which is widely expressed in human body (Falk *et al.*, 2008; Murata et al., 2016; Huang et al., 2018). Of note, KCNJ4 is up-regulated by the stimulation of epidermal growth factor receptor (EGFR) that was required for multiple malignancies (Zhang D et al., 2011). In our previous research, we found that high-regulation of KCNJ4 is related with LADC progression and poor prognosis of LADC patients(Wu X and Yu, 2019). However, the underlying mechanism of how KCNJ4 regulates the progression of LADC still remains unclear. MicroRNAs (miRNAs) are a small non-coding RNAs that bind with the 3’-untranslated region (3’-UTR) of key genes to modulate various cancers (Liao et al., 2013; Jin et al., 2019). Therefore, we used bioinformatics tools to screen the potential miRNAs that might target KCNJ4 and discovered that miR-34c-5p might be an upstream miRNA of KCNJ4 in LADC. Several publications have indicated that miR-34c-5p can influence the development of laryngeal squamous cell carcinoma (Re and Magliulo, 2017), and the resistance of lung cancer (Catuogno *et al.*, 2013).

Correlations among long non-coding RNAs (lncRNAs), miRNAs and genes have recently emerged as a hot research field in tumor biological processes (Cheng et al., 2019; Wang A et al., 2019). LncRNAs, a family of RNA molecules longer than 200 base pairs without coding partial (Batista and Chang, 2013), have been identified as crucial factors in multiple biological processes, such as cell growth, differentiation and apoptosis (Nagano and Fraser, 2011; Marchese et al., 2017). In a variety of cancers, lncRNAs are involved in a broad range of molecular mechanisms containing functioning as ceRNAs, scaffolds and signals to regulate the expression of gene (Tsai et al., 2010; Wang K and Chang, 2011). Numerous publications have elaborated that lncRNAs, served as competitive endogenous RNAs (ceRNAs), can directly sponge miRNAs and regulate key genes, thereby modulating the processes of physiological and pathological in human (Li et al., 2019; Tian et al., 2019; Zhang Y et al., 2019). Thus, we then screened the possible lncRNAs of miR-34c-5p and KCNJ4 to form a ceRNA mechanism. Our bioinformatics prediction and comprehensive analysis suggested that POU6F2-AS1 (POU6F2-*antisense* 1) might act as a ceRNA candidate for miR-34c-5p in LADC. Although the role of POU6F2-AS1 in tumors still remains unclear, POU6F2-AS2 (POU6F2-*antisense* 2; the homology of POU6F2-AS1) has been reported to be associated with Ybx1 protein and participates in the chromatin localization of Ybx1 to specially regulate the prognosis of esophageal squamous cell carcinoma (ESCC) (Liu J et al., 2016).

We performed this study to verify the correlation between POU6F2-AS1, miR-34c-5p and KCNJ4 and determine the biological role of POU6F2-AS1-miR-34c-5p-KCNJ4 network in LADC development. Our results illustrated that POU6F2-AS1 was highly expressed in LADC and identified as a ceRNA of miR-34c-5p to regulate KCNJ4. Moreover, our results revealed that POU6F2-AS1 contributed to LADC proliferation and invasion via modulating miR-34c-5p/KCNJ4 axis.

## Material and Methods

### Collection of human clinical specimens

TCGA database provided LADC clinical cases and adjacent normal samples for the analysis of POU6F2-AS1 (57 pairs) and miR-34c-5p (46 pairs) expression. Additional, 471 LADC samples with complete clinical data were selected to plot the overall survival curve of LADC patients by Kaplan-Meier method based on the TCGA portal. GSE74190, a total of 44 normal lung tissues and 36 human LADC tissues, was obtained from GEO database to assess the expression of miR-34c-5p in LADC tissues.

### Cell lines and transfection

All LADC cell lines consisting of Calu-3, A549, NCI-H209 and NCI-H460, and human normal lung epithelial cell line BEAS2B were purchased from Cell Biology of the Chinese Academy of Sciences (Shanghai, China). They were incubated in Dulbecco’s modified Eagle’s medium (DMEM) consisting of 10 % fetal bovine serum (FBS), 100 U/mL penicillin and 0.1 mg/mL streptomycin at 37 ℃ with 5 % CO_2_.

Transient transfection was performed by Lipofectamine 2000 in accordance with the manufacturer’s protocols. The following agents synthesized by GenePharma Co., Ltd (Shanghai, China) were utilized in this study: si-POU6F2-AS1#1 (5’-TGCAGAACCTGACC-3’), si-POU6F2-AS1#2 (5’-CCGAGAAGTAGTTA-3’), si-KCNJ4 (5’-AAGGTGGACTACTCACGT-3’), si-con (5’-CGAACUCACUGGUCUGACC-3’), pcDNA3.1-POU6F2-AS1, pcDNA3.1-KCNJ4 and miR-34c-5p mimic/inhibitor.

### qRT-PCR assay

LADC cells were lysed and whole RNA was isolated by TRIzol reagent based on of the manufacturer’s instructions. The RNA of POU6F2-AS1 and KCNJ4 were reverse transcribed into complementary DNA (cDNA) utilizing PrimeScript RT kit (Takara biomedical Technology Co., Ltd., Beijing, China). And SYBR Premix Ex Taq II (Takara biomedical Technology Co., Ltd.) was used to examine the expression of POU6F2-AS1 and KCNJ4 on 7500HT real-time PCR system. The cDNA of miR-34c-5p was generated using MiScript Reverse Transcription kit (Qiagen, Shanghai, China) and its expression was measured by MiScript SYBR-Green PCR kit (Qiagen). Primers required for this investigation were as follows: POU6F2-AS1 F: 5’-TCCACTAGCAAGTCAGGCTGCA-3’, R: 5’-GCATCAGTGGAATGGTCCCGAT-3’; miR-34c-5p F: 5’-GGCAGTGTAGTTAGCTG -3’, R: 5’-GAACATGTCTGCGTATCTC-3’; U6 F: 5’-CTCGCTTCGGCAGCACATATACT-3’, R: 5’-ACGCTTCACGAATTTGCGTGTC-3’; KCNJ4 F: 5’-CGAGGAGAAGAGCCACTACAAG-3’, R: 5’-GTTCTCGTAGCAGAAGGCACTG-3’; GAPDH F: 5’-TGTGTCCGTCGTGGATCTGA-3’, R: 5’-CCTGCTTCACCACCTTCTTGA-3’. For the detection of POU6F2-AS1 and KCNJ4, GAPDH was considered as the internal control; for the detection of miR-34c-5p, U6 was considered as the internal control.

### Western blotting

RIPA buffer with protease inhibitor was employed to extract protein from transfected cells, and then the concentration of protein was quantified with BCA method. Equal amount of protein (20 μg) was electrophoresised in 12 % SDS-PAGE and transferred to PVDF membranes. Next, 5 % skimmed milk powder was utilized to block the PVDF membranes for 1 h at 25 ℃, and at 4 ℃, the membranes were incubated with primary antibodies against KCNJ4 (PA5-39601; Gibco, Thermo Fisher Scientific Inc, Waltham, MA) and GAPDH (39-8600; Thermo Fisher Scientific Inc) overnight. After washed with PBS, secondary antibody was applied to incubate PVDF membranes at room temperature for 1 h. Protein bands were developed using enhanced chemiluminescence and scanned with QUANTITY ONE software.

### CCK-8 analysis

Forty-eight hours after transfection, 1000 cells/well were plated into 96-well plates and cultured at 37 ℃ for different time spots (0, 24, 48 and 72 h). 10 μL of CCK-8 reagents were added into each well to culture cells for 1.5 h. Finally, the optical density value at 450 nm was assessed by the microplate reader and the curve of cell proliferation was plotted in accordance with the optical density value at each time spot.

### Colony formation assay

After 48 h transfection, LADC cells (500 cells) were seeded on a 60 mm dish and incubated in DEME medium with 10 % FBS at 37 ℃ with 5 % CO_2_ for two weeks. When macroscopic colonies appeared in the culture dish, PBS was utilized to wash colonies for three times, and 4 % paraformaldehyde and 0.1 % crystal violet were used to fix and stain colonies respectively. Afterwards, colonies were photographed and the number of colonies was quantified by Image J.

### Transwell invasion and migration assays

The 24-well transwell chamber with an aperture of 8 μm was pre-coated with Matrigel and utilized to assess the invasive potential of LADC cells. Transfected LADC cells (1 × 10^5^) in the serum-free medium were put into the upper chamber and 500 μL of DEME medium with 10 % FBS was placed into the lower chamber. Followed by 24 h incubation, non-invasive cells on the upper chamber were removed using cotton swabs. Invasive cells on the lower chamber were fixed with 4% paraformaldehyde and dyed by 0.1 % crystal violet. Finally, invasive cells from five randomly fields were pictured under a microscope and counted using Image J. Transwell migration assay was similar to the invasion assay, and the transwell chamber did not need to be pre-coated with Matrigel and the inoculated density was 5 × 10^3^.

### Luciferase activity analysis

Luciferase activity analysis was performed to examine whether miR-34c-5p interacts with POU6F2-AS1 and KCNJ4 3’UTR, sequences of POU6F2-AS1 and KCNJ4 3’UTR including wild-type (WT)/mutant (MUT) miR-34c-5p putative binding sites were cloned into pGL3-Luc vector and generated WT-POU6F2-AS1, MUT-POU6F2-AS1, WT-KCNJ4 and MUT-KCNJ4. For the detection of luciferase activity, HEK 293T cells were co-transfected with the above mentioned vectors and miR-34c-5p mimic or miR-34c-5p mimic NC by Lipofectamine 2000. At 48 h post transfection, luciferase reporter assay kit (Promega, Madison, WI, USA) was applied to determine the relative luciferase activity.

### Statistical analysis

All data were presented as means ± standard deviation (SD) and each experiment was repeated in three times. Statistical analyses were analyzed using SPSS 22.0 and graphed with GraphPad Prism 5.0. Pearson’s correlation analysis was applied to verify the relationship between KCNJ4 expression and POU6F2-AS1 or miR-34c-5p in both LADC tissues and cells. All comparisons of this study were compared using Student’s t-test or one-way analysis of variance with Dunnett’s or Bonferroni’s post hoc test. P < 0.05 was regarded as statistically significance.

### Data availability statement

The data in this study is available from the corresponding author on reasonable request.

## Results

### MiR-34c-5p expression is associated with KCNJ4 and POU6F2-AS1 in LADC

**Figure 1:**
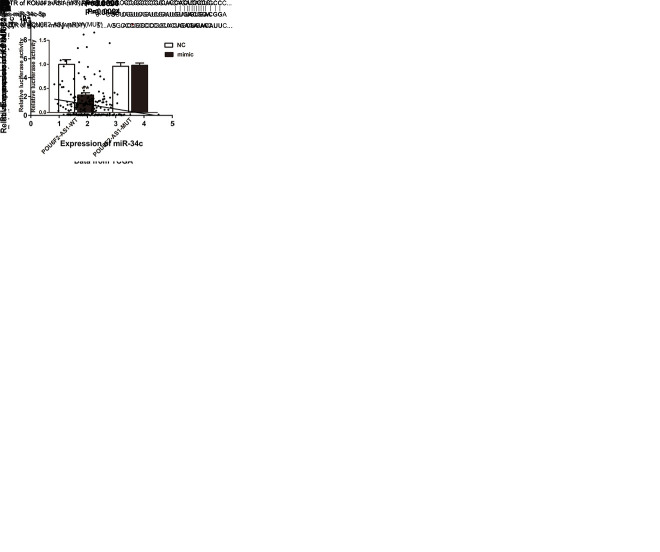
Correlations between miR-34c-5p and POU6F2-AS1 or KCNJ4 in LADC and relative expression level of POU6F2-AS1 in LADC tissues and cell lines, and its prognostic significance. Relative expression level of miR-34c-5p in LADC tissues and normal tissues based on (A) GEO dataset (44 normal tissues and 36 LADC tissue samples) and (B) TCGA database (46 pairs of LADC tissues and normal samples). Dual-luciferase reporter assays were performed to test the relevance of miR-34c-5p and (C) POU6F2-AS1 and (D) KCNJ4. Pearson’s correlation analysis was used to detect the relationship between KCNJ4 and (E) miR-34c-5p or (F) POU6F2-AS1. (G) Relative expression level of POU6F2-AS1 was determined in LADC tissues (n = 57) compared with adjacent normal tissues (n = 57), P < 0.0001. (H) Overall survival curve of LADC patients with high and low POU6F2-AS1 expression levels, P = 0.018. (I) Relative expression level of POU6F2-AS1 in four LADC cell lines and one human normal lung epithelial cell line BEAS2B, **P < 0.01.

Our previous publication demonstrated that KCNJ4 was significantly increased in LADC tissues and cells, and was associated with unfavorable prognosis of LADC patients (Wu and Yu, 2019). Therefore, we predicted the upstream miRNAs of KCNJ4 using miRanda, miRWalk and TargetScan for further the detection of potential mechanism. We intersected the 29 putative miRNAs with the 17 down-regulated miRNAs obtained from the GEO database and finally only obtained miR-34c-5p. Analysis of GSE74190 array and TCGA-LADC cohort indicated that miR-34c-5p was decreased in LADC tissues compared with normal tissues (Figure 1A, B, P < 0.05). Then, we screened the possible upstream lncRNAs of miR-34c-5p on the basis of online prediction (LncBase v.2), a total of 167 upstream lncRNAs were achieved. After intersecting possible upstream lncRNAs with up-regulated differentially expressed genes from TCGA database, 14 genes were ultimately obtained, including POU6F2-AS1. Subsequently, comprehensive literature and prognostic anlyses, POU6F2-AS1 was selected to further analyze.

To verify the correlations between KCNJ4 and miR-34c-5p or POU6F2-AS1, dual-luciferase reporter assays were implemented. Results illustrated that luciferase activity of POU6F2-AS1-WT was markedly reduced by miR-34c-5p mimic, whereas miR-34c-5p mimic NC had no influence on POU6F2-AS1-WT luciferase activity (Figure 1C, P < 0.01). Furthermore, the luciferase activity of KCNJ4-WT was also inhibited by miR-34c-5p mimic and miR-34c-5p mimic NC didn’t affect the luciferase activity (Figure 1D, P < 0.01). In addition, Pearson’s analysis based on the TCGA database showed that miR-34c-5p expression was inversely proportional to KCNJ4 expression, while POU6F2-AS1 was proportional to KCNJ4 (Figure 1E, F, P < 0.05). Their correlations have also been identified in LADC cells (Calu-3 and NCI-H460 cells) using Pearson’s correlation analysis, which consistent with that in LADC tissue samples (Figure S1). Collectively, miR-34c-5p may be related with KCNJ4 and POU6F2-AS1 in LADC.

### POU6F2-AS1 is significantly increased in LADC and correlated with unfavorable prognosis

By accessing the TCGA database, we found that POU6F2-AS1 was highly regulated in LADC tissues (n = 57) compared with adjacent normal tissues (n = 57; Figure 1G, P < 0.0001). To determine the correlation between POU6F2-AS1 expression and prognosis of LADC patients, 471 LADC patients were divided into high (> median value of POU6F2-AS1 expression) and low (< median value of POU6F2-AS1 expression) POU6F2-AS1 expression groups. Kaplan-Meier analysis showed that the expression level of POU6F2-AS1 was associated with prognosis of LADC patients: patients (n = 236) with high-regulated POU6F2-AS1 expression had poorer outcome than patients (n = 235) with low-regulated POU6F2-AS1 expression (Figure 1H, P = 0.018). Moreover, qRT-PCR was performed to assess the expression of POU6F2-AS1 in four LADC cell lines (Calu-3, A549, NCI-H209 and NCI-H460) and human normal lung epithelial cell line (BEAS2B). As shown in Figure 1I, results of qRT-PCR indicated that the expression of POU6F2-AS1 in all LADC cell lines was higher than normal lung epithelial cell line (P < 0.01). According to the highest POU6F2-AS1 expression of Calu-3 and lowest POU6F2-AS1 expression of NCI-H460, we selected Calu-3 and NCI-H460 cell lines for the future experiments (Figure 1I, P < 0.01).

### POU6F2-AS1 boosts the proliferation, colony formation, invasion and migration of Calu-3 and NCI-H460 cells

**Figure 2:**
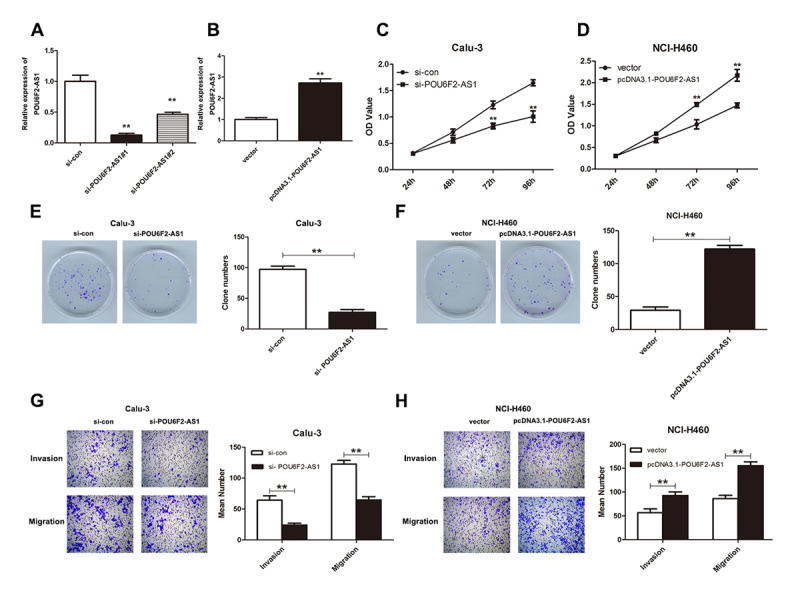
Effects of POU6F2-AS1 on aggressiveness of Calu-3 and NCI-H460 cells. (A) The expression level of POU6F2-AS1 in Calu-3 cells transfected with si-con, si-POU6F2-AS1#1 and si-POU6F2-AS1#2. (B) QRT-PCR showed the expression level of POU6F2-AS1 after pcDNA3.1-POU6F2-AS1 transfection. CCK-8 assay were conducted to assess the effect of POU6F2-AS1 on proliferation of (C) Calu-3 and (D) NCI-H460 cells. Role of POU6F2-AS1 was revealed using (E) Calu-3 and (F) NCI-H460 cells in colony formation assays. (G) Calu-3 and (H) NCI-H460 cells were utilized to examine the biological influence of POU6F2-AS1, **P < 0.01.

To ascertain the functional role of POU6F2-AS1 in LADC cells, si-con, si-POU6F2-AS1#1 and si-POU6F2-AS1#2 were transfected into Calu-3 cells, and pcDNA3.1 empty vector and pcDNA3.1-POU6F2-AS1 were transfected into NCI-H460 cells. qRT-PCR analyses demonstrated that POU6F2-AS1 expression was down-regulated in Calu-3 cells after transfected with si-POU6F2-AS1#1 and si-POU6F2-AS1#2 relative to Calu-3 cells transfected with si-con (Figure 2A, P < 0.01). By contrast, POU6F2-AS1 was overexpressed in NCI-H460 cells transfected with pcDNA3.1-POU6F2-AS1 (Figure 2B, P < 0.01).

Next, we conducted functional experiments in Calu-3 and NCI-H460 cells. CCK-8 assay revealed that down-regulation of POU6F2-AS1 inhibited proliferation of Calu-3 cells (Figure 2C, P < 0.01), whilst overexpression of POU6F2-AS1 improved proliferative ability of NCI-H460 cells (Figure 2D, P < 0.01). Knockdown of POU6F2-AS1 suppressed the clonogenic potential of Calu-3 cells (Figure 2E, P < 0.01), while colony formation of NC-I-H460 cells was promoted because of POU6F2-AS1 overexpression (Figure 2F, P < 0.01). Similarly, transwell invasion and migration assays indicated that the number of invasive and migratory cells was decreased after POU6F2-AS1 knockdown; when we up-regulated POU6F2-AS1, the opposite result was achieved (Figure 2G, H, P < 0.01). Collectively, these findings suggested that POU6F2-AS1 can strengthen cell proliferation, colony formation, invasion and migration in LADC cells.

### POU6F2-AS1 serves as a ceRNA via sponging miR-34c-5p to elevate KCNJ4

**Figure 3:**
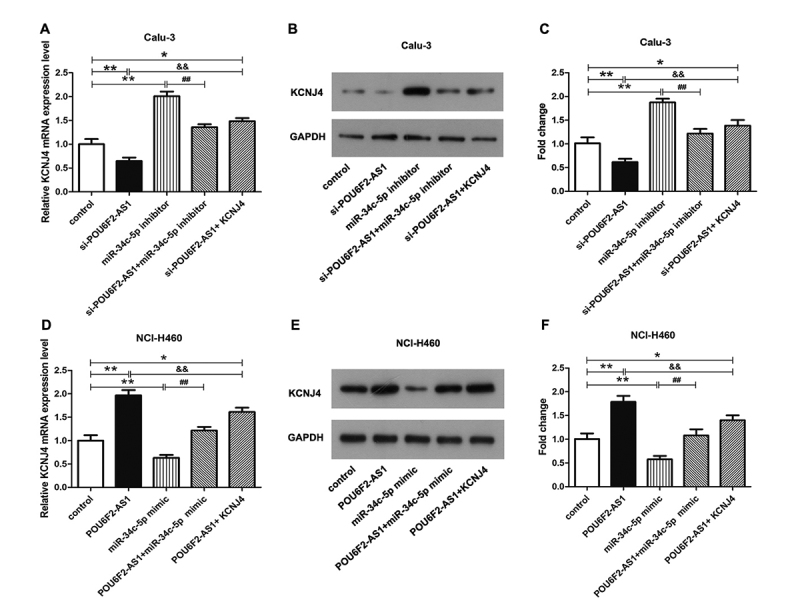
POU6F2-AS1 served as a ceRNA via sponging miR-34c-5p to elevate KCNJ4. (A) The mRNA and (B) protein expression level of KCNJ4 in Calu-3 cells were demonstrated. (C) The protein expression level of KCNJ4 was quantified. (D) The mRNA and (E) protein expression level of KCNJ4 in NCI-H460 cells were detected. (F) The quantification of (H). *P < 0.05, **P < 0.01, ^##^P < 0.01 and ^&&^P < 0.01.

qRT-PCR and western blotting revealed that POU6F2-AS1, as a potential ceRNA of miR-34c-5p, can reverse the effect of miR-34c-5p on KCNJ4. In Calu-3 cells, we found that si-POU6F2-AS1 inhibited the expression of KCNJ4 and miR-34c-5p inhibitor elevated KCNJ4 expression, and the promoting effect of miR-34c-5p inhibitor on KCNJ4 expression was weakened by si-POU6F2-AS1 (Figure 3A-C, P < 0.05). As expected, overexpression of POU6F2-AS1 facilitated the expression of KCNJ4, whereas miR-34c-5p mimic blocked KCNJ4 expression. Moreover, the inhibitory effect of miR-34c-5p mimic was reversed by overexpression of POU6F2-AS1 (Figure 3D-F, P < 0.05). Taken together, all results showed that POU6F2-AS1 served as a ceRNA via sponging miR-34c-5p to elevate KCNJ4.

### Effects of POU6F2-AS1 on LADC cell behaviors are achieved by miR-34c-5p/KCNJ4 axis

**Figure 4:**
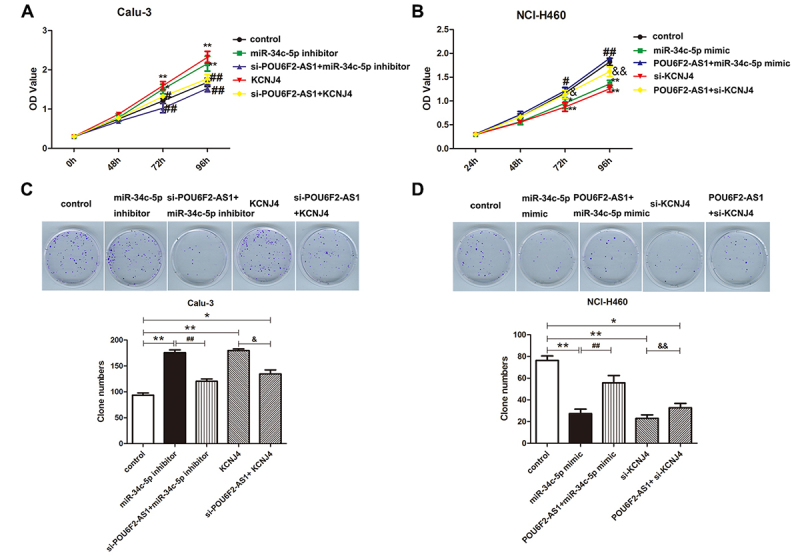
Effect of POU6F2-AS1 on cell proliferation and colony formation in LADC cells was reversed by miR-34c-5p via targeting KCNJ4. (A and B) CCK-8 and (C and D) colony formation assay were implemented to investigate proliferative and colongenic capabilities, respectively. *P < 0.05, **P < 0.01, ^##^P < 0.01 and ^&^P < 0.05.

**Figure 5:**
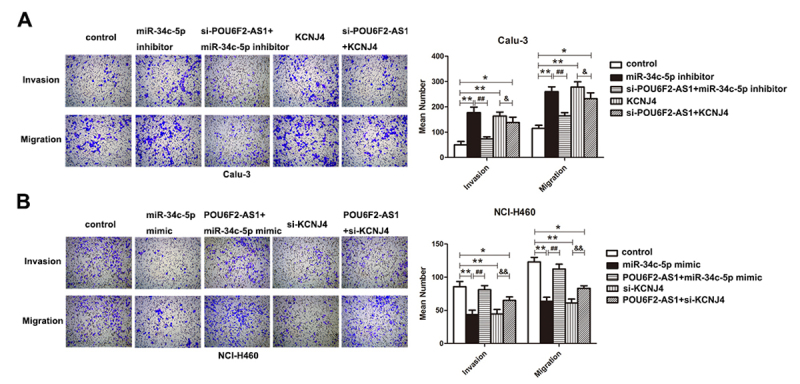
Effect of POU6F2-AS1 on invasion and migration of LADC cells was recovered by miR-34c-5p via targeting KCNJ4. (A) and (B) Transwell experiments were performed utilizing Calu-3 and NCI-H460 cells. *P < 0.05, **P < 0.01, ^##^P < 0.01 and ^&&^P < 0.01.

To further investigate whether miR-34c-5p can reverse the effect of POU6F2-AS1 on cell aggressiveness, rescue experiments *in vitro* were performed. miR-34c-5p knockdown and overexpression of KCNJ4 promoted cell behaviors, including proliferation, colony formation, invasion and migration (Figure 4A, C and 5A, P < 0.05). Interestingly, co-transfection with si-POU6F2-AS1 and miR-34c-5p inhibitor in Calu-3 cells reversed the promoting influence of miR-34c-5p inhibitor, and co-transfection with si-POU6F2-AS1 and overexpression of KCNJ4 indicated that si-POU6F2-AS1 attenuated the promoting influence of high-regulated KCNJ4. In NCI-H460 cells, cell aggressiveness was impaired by miR-34c-5p mimic and si-KCNJ4 (Figure 4B, D and 5B, P < 0.05). Co-transfection with overexpression of POU6F2-AS1 and miR-34c-5p mimic revealed that overexpression of POU6F2-AS1 recovered the inhibitory effect of miR-34c-5p mimic; co-transfection with overexpression of POU6F2-AS1 and si-KCNJ4 suggested that high-regulation of POU6F2-AS1 relieved si-KCNJ4’s suppressive effect on cell behaviors. In total, the effect of POU6F2-AS1 on LADC cell behaviors may be correlated with miR-34c-5p/KCNJ4.

## Discussion

This present study detected the expression level of POU6F2-AS1 in LADC, which correlated with prognosis of LADC patients. Results of functional experiments illuminated that POU6F2-AS1 can modulate the proliferation, colony formation, invasion and migration of LADC cells by regulating miR-34c-5p and KCNJ4, thereby mediating the development of LADC.

As detailed in our previous study, overexpression of KCNJ4 is closely linked with poor prognosis of LADC patients, and reduction of KCNJ4 inhibits the proliferation, migration and invasion of LADC cells via the MEK/ERK signaling pathway (Wu and Yu, 2019). To determine the potential molecular mechanism of KCNJ4-related network in LADC, online prediction was conducted to predict the potential miRNAs that were linked with KCNJ4. Analysis indicated that miR-34c-5p might serve as an upstream miRNA of KCNJ4. GSE74190 array and TCGA-LADC cohort showed that miR-34c-5p was down-regulated in LADC tissues, and luciferase reporter assay and Pearson’s analysis verified the association between miR-34c-5p and KCNJ4. In addition, extensive literature reports have shown that miR-34c-5p is correlated with cell proliferation, migration, invasion and apoptosis in cervical cancer (Cordova-Rivas et al., 2019), colorectal cancer (Wan et al., 2017) and glioma (Wu Z *et al.*, 2013), suggesting that miR-34c-5p might be required for various cancer progression. Xu Z *et al*. (2019) determined that lncRNA HCG18/miR-34c-5p/NOTCH1 network can regulate the proliferation and migration of bladder cancer cells. Of note, Wang Y *et al*. (2019) discovered that miR-34c-5p, FAM83A, FLJ26245 and KCNQ1OT1 are closely correlated with the survival rates of LADC patients. These analyses and previous studies manifested that the miR-34c-5p/KCNJ4 pair might participate in LADC development.

Previous studies have shown that many lncRNAs hold the capability to regulate cancer progression by altering gene expression and binding with different types of miRNAs, such as nuclear enriched abundant transcript 1(NEAT1) in breast cancer (Pang et al., 2019), colon cancer-associated transcript 2 (CCAT2) in osteosarcoma (Liu J et al., 2019) and homeobox D gene cluster antisense growth-associated long noncoding RNA (HAGLR) in esophageal cancer (Yang et al., 2019). The question of how lncRNAs modulate LADC malignancy is gaining increasing tremendous attention. We predicted possible upstream lncRNAs of miR-34c-5p using LncBase v.2 and obtained 167 potential lncRNAs. After intersecting these predicted lncRNAs with up-regulated DEGs, a total of 14 lncRNAs were retrieved, including POU6F2-AS1. Through comprehensive literature and prognostics analysis, POU6F2-AS1 was identified as a candidate ceRNA to sponge miR-34c-5p. POU6F2-AS1 is a key regulator in tumorigenesis located in chromosome 7p14.1. According to the report by Mattick *et al.* (2009), lncRNAs are composed of five subgroups: (1) sense (2) antisense (3) bidirectional (4) intronic (5) intergenic, which would be defined on the basis of their position. Antisense lncRNAs have been considered as powerful tools for the regulation of X-chromosome inactivation, genomic imprinting and development of diverse diseases (Xu J et al., 2018). POU6F2-AS1 has been classified as an antisense lncRNA. Although the role of POU6F2-AS1 in tumors still remains unclear, many antisense lncRNAs have been shown to play crucial roles in a wide range of cancers, including LADC. A prior investigation performed by Xue *et al*. (2019) demonstrated that lncRNA ZFPM2-AS1 facilitates cell proliferation via miR-18b-5p/VMA21 axis in LADC (Xue *et al.*, 2019). Cisplatin resistance of LADC cells is driven by the network of lncRNA HOXA11-AS/miR-454-3p/Stat3 (Zhao et al., 2018). Moreover, lncRNA MUC5B-AS1 is involved in metastasis through mediating MUC5B expression in LADC (Yuan et al., 2018) as well as lncRNA TP73-AS1 (Liu C *et al.*, 2019). Given these reports, we found that up-regulation of POU6F2-AS1 in LADC tissues was linked with unfavorable prognosis of LADC patients. Loss-of-function and gain-of-function determined that POU6F2-AS1 facilitated aggressiveness of LADC cells. Moreover, rescue experiments elucidated that miR-34c-5p/KCNJ4 axis was involved in the regulation of POU6F2-AS1 to LADC cells. All data indicated that POU6F2-AS1-miR-34c-5p-KCNJ4 network might serve as a viable therapeutic therapy for LADC in the future.

## Conclusion

The POU6F2-AS1-miR-34c-5p-KCNJ4 network was proposed in this present study. POU6F2-AS1 expression was significantly increased in LADC tissues and cells. Its high-regulation was associated with unfavorable prognosis of LADC patients. Functional *in vitro* experiments disclosed that POU6F2-AS1 can affect the proliferative, clonogenic, migratory and invasive abilities of LADC cells. Furthermore, the negative relationship between miR-34c-5p and POU6F2-AS1, as well as positive correlation between KCNJ4 and POU6F2-AS1, were further determined via rescue analyses. Taken together, the discovery of the POU6F2-AS1-miR-34c-5p-KCNJ4 network might contribute to LADC development, indicating its potential prognostic significance and potentially driving new therapeutic strategies may be produced to treat LADC.

## Supplementary material

The following online material is available for this article:

Figure S1 - miR-34c-5p expression was inversely proportional to KCNJ4, while POU6F2-AS1 was positively proportional to KCNJ4 in LADC cells.
