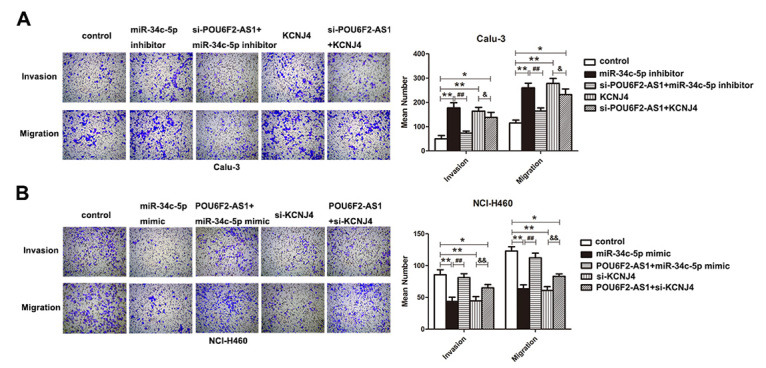# Erratum: Long noncoding RNA POU6F2-AS1 regulates lung cancer aggressiveness through sponging miR-34c-5p to modulate KCNJ4 expression

**DOI:** 10.1590/1678-4685-GMB-2020-0050er

**Published:** 2022-08-15

In the article “Long noncoding RNA POU6F2-AS1 regulates lung cancer aggressiveness through sponging miR-34c-5p to modulate KCNJ4 expression”, with DOI number: 10.1590/1678-4685-GMB-2020-0050, published in the journal Genetics and Molecular Biology, 44(2):e20200050, the authors noted an error in Figure 5, on page 6 of the article. They reported the inclusion of a wrong microscopy image in Figure 5A for the Invasion assay of the si-POU6F2-As1+KCNJ4 group, and another wrong image for the Migration result of the Control group shown in Figure 5B for the NCI-H460 cells. The author provided a new Figure 5, which is now published together with this Erratum note. The error has no consequences for the statistical analysis of the results of this experiment and has no effect on the conclusions drawn and presented in this article.