# Supplementary material for: Long noncoding RNA POU6F2-AS1 regulates lung cancer aggressiveness through sponging miR-34c-5p to modulate KCNJ4 expression
Source: Genet Mol Biol. 2021 May 14;44(2):e20200050. doi: 10.1590/1678-4685-GMB-2020-0050 (PMC8127722; doi:10.1590/1678-4685-GMB-2020-0050)

Genetics and Molecular Biology
DOI: https://doi.org/10.1590/1678-4685-GMB-2020-0050

Supplementary material to "Long noncoding RNA POU6F2-AS1 regulates lung cancer aggressiveness through sponging miR-34c-5p to modulate KCNJ4 expression"

The following online material is available for this article:

Figure S1 - miR-34c-5p expression was inversely proportional to KCNJ4, while POU6F2-AS1 was positively proportional to KCNJ4 in LADC cells. (A-C) qRT-PCR analysis was performed to detect the expression of POU6F2-AS1, KCNJ4 and miR-34c-5p in BEAS2B, Calu-3 and NCI-H460 cell lines, **P < 0.01. (D-G) Pearson's correlation analysis.

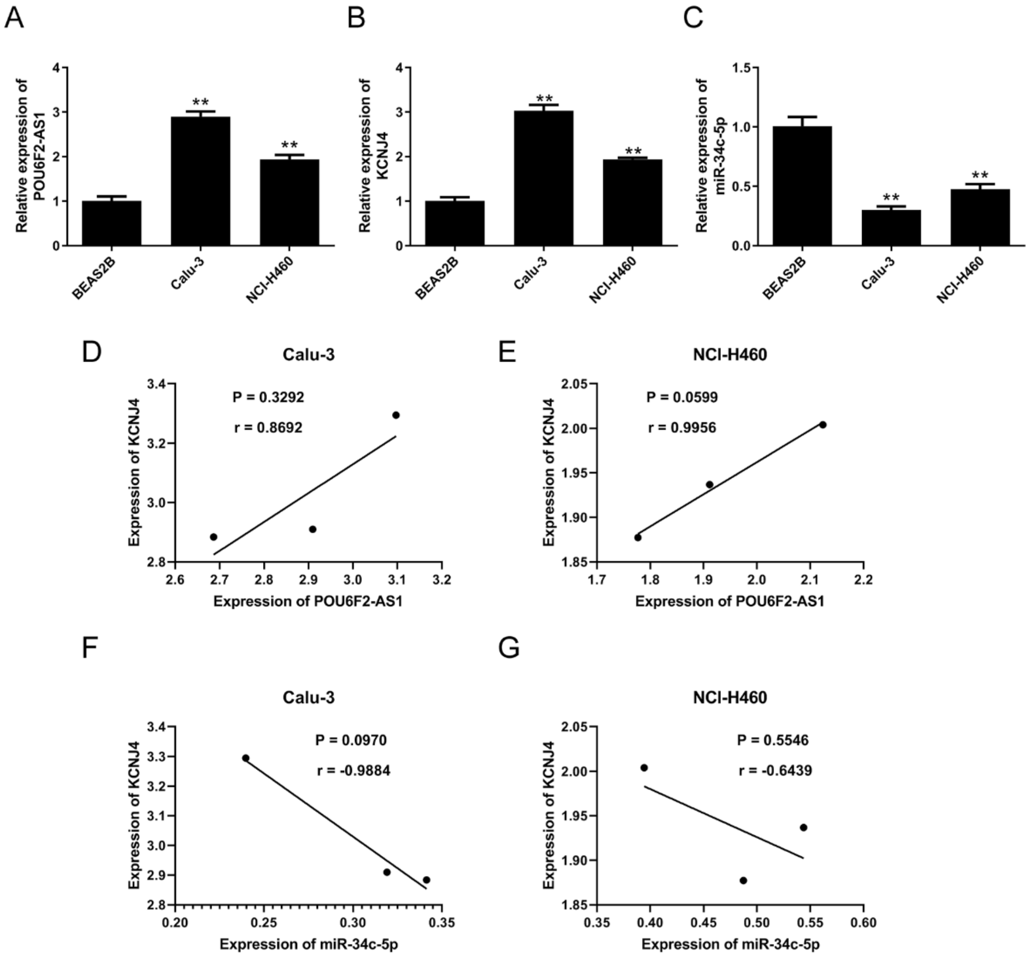

Supplement: Figure S1 - [file 1415-4757-GMB-44-2-e20200050-s1.pdf]
